# Effect of Dietary Organic Acids and Botanicals on Metabolic Status and Milk Parameters in Mid–Late Lactating Goats

**DOI:** 10.3390/ani13050797

**Published:** 2023-02-22

**Authors:** Andrea Giorgino, Federica Raspa, Emanuela Valle, Domenico Bergero, Damiano Cavallini, Marta Gariglio, Valentina Bongiorno, Giorgia Bussone, Stefania Bergagna, Francesca Cimino, Lucrezia Dellepiane, Gilberto Mancin, Richard Paratte, Víctor Sáinz de la Maza-Escolà, Claudio Forte

**Affiliations:** 1 Department of Veterinary Sciences, University of Turin, 10095 Grugliasco, Italy; 2 Department of Veterinary Sciences, University of Bologna, 40064 Ozzano dell’Emilia, Italy; 3 Istituto Zooprofilattico Sperimentale del Piemonte, Liguria e Valle d’Aosta, 10154 Torino, Italy; 4 Azienda Sanitaria Locale Novara, ASL NO, 28100 Novara, Italy; 5 Vetagro S.p.A., 42124 Reggio Emilia, Italy

**Keywords:** dairy goats, nutrition, sustainable farming, supplementation, blood profile, milk performance

## Abstract

**Simple Summary:**

Alternative feed additives could offer nutritional strategies that help to prevent metabolic disorders in ruminants by exerting beneficial effects on the animals’ metabolic and immune statuses. In particular, organic acids (OA), such as citric acid and sorbic acid, and pure botanicals (PB), such as thymol and vanillin, have been widely used in the field of animal nutrition for their positive effects on production performances and known effects on metabolic and immune statuses. Since no information is available on goats, the goal of this study was to investigate the effects of OA/PB supplementation on the metabolic status and milk parameters of dairy goats. The results showed that the metabolic status of the treated goats was not negatively affected by the OA/PB supplementation. Moreover, OA/PB increased the milk fat content and the milk coagulation index, which are favorable effects in relation to the technological properties of milk. These findings encourage further studies on OA/PB supplementation in the dairy goat diet.

**Abstract:**

The microencapsulated mixture of organic acids and pure botanicals (OA/PB) has never been evaluated in goats. The aim of this study was to extend the analysis to mid–late lactating dairy goats, evaluating the effects of OA/PB supplementation on the metabolic status, milk bacteriological and composition characteristics, and milk yield. Eighty mid–late lactating Saanen goats were randomly assigned to two groups: one group was fed the basal total balanced ration (TMR) (CRT; *n* = 40) and the other was fed a diet that was TMR supplemented with 10 g/head of OA/PB (TRT; *n* = 40) for 54 days during the summer period. The temperature–humidity index (THI) was recorded hourly. On days T0, T27, and T54, the milk yield was recorded, and blood and milk samples were collected during the morning milking. A linear mixed model was used, considering the fixed effects: diet, time, and their interaction. The THI data (mean ± SD: 73.5 ± 3.83) show that the goats did not endure heat stress. The blood parameters fell within the normal range, confirming that their metabolic status was not negatively influenced by OA/PB supplementation. OA/PB increased the milk fat content (*p* = 0.04) and milk coagulation index (*p* = 0.03), which are effects that are looked on as favorable by the dairy industry in relation to cheese production.

## 1. Introduction

One aim of the livestock industry is to maximize productivity, while keeping antibiotic usage to a minimum to achieve more sustainable and higher quality production [1,2]. Alternative feed additives, such as dietary acidifiers, essential oils, probiotics, and prebiotics, have been introduced as potential replacements for antibiotics and as nutritional strategies to prevent metabolic disorders in ruminants [3]. Of the possible feed additives, organic acids (OA) such as citric acid and sorbic acid and pure botanicals (PB) such as thymol and vanillin have been widely used in the field of animal nutrition for their positive effects on production performances and the metabolic and immune statuses [4,5,6]. The moderate-to-strong antimicrobial activity of OA occurs thanks to their ability to penetrate the bacterial cell wall and inhibit microbial enzymatic reactions and nutrient transport systems [7]. The antioxidant and anti-inflammatory activities exerted by PB also occur through their capacity to impair the enzymatic systems involved in bacterial energy production and structural component synthesis [8,9]. The PB vanillin is often used as a flavoring agent to improve the feed palatability [10]. The specific combination of OA (citric and sorbic acids) and PB (thymol and vanillin) as a microencapsulated mixture (16.7% sorbic acid, 25% citric acid, 1.7% thymol, and 1% vanillin) within a slow-release lipid matrix (OA/PB) has already been shown to exert antimicrobial, anti-inflammatory, antioxidant, and immunomodulatory properties in, for example, supplementing the feed of weaned pigs with OA/PB improved growth performances and intestinal integrity by modulating inflammatory processes at both the local and systemic levels [11]. A modulatory effect on the immune status was also shown in broiler chickens [12]. The authors showed how dietary supplementation with this specific microencapsulated compound led to immunological benefits by increasing the in vitro activity of the peripheral blood leukocytes, as well as the heterophil and monocyte counts. Moreover, in a study carried out by Fontoura and colleagues [6], giving the same microencapsulated product to heat-stressed Holstein dairy cows was associated with improvements in the milk yield, the energy-corrected milk value, and milk nitrogen efficiency. Most of the available scientific literature explores the immunomodulatory and antioxidant properties of OA/PB, whereas, to our best knowledge, the information on OA/PB supplementation on metabolic status and milk performances in ruminants is still sparse. Therefore, the aim of the present study was to evaluate the effects of the dietary inclusion of this specific microencapsulated product containing OA/PB on the metabolic status (hematological and hematochemical parameters) and milk (milk yield, bacteriological characteristics, and milk composition) of mid–late lactation goats. We hypothesized that OA/PB supplementation may also have beneficial effects on the goats’ metabolic status, as well as the milk yield and quality during the mid–late lactating phase.

## 2. Materials and Methods

The experimental protocol was designed according to the guidelines of the current European Directive on the care and protection of animals (2010/63/EU), and it was approved by the Ethical Committee of the Department of Veterinary Sciences of the University of Turin (Italy, Prot. N. 0002184/2021).

### 2.1. Animals, Management, and Environmental Conditions

The present study was conducted on a breeding farm that rears around 250 Saneen goats for milk production in north-west Italy. A total of 80 homogenous and clinically healthy Saanen goats in the mid–late lactation phase (110 days in milk), mean ± SD BCS of 3.4 ± 0.03 on a 5-point scale [13], were involved in the study. The feeding trial started at T0, corresponding to 105 days from the beginning of lactation, and lasted for 54 days (T54). The health status of the animals was monitored on a daily basis throughout the entire feeding trial (from T0 to T54) by a veterinarian expert in small ruminants. At T0, the dairy goats were randomly divided into two adjacent group pens, each covering an area of 80 m^2^. The two group pens were separated from each other by the central feeding lane and located inside a naturally ventilated shed that was open on two sides. The concrete floor was covered with a layer of wheat straw bedding. The animals had ad libitum access to tap water. Since the study was carried out in the summer period (July–August), a single data logger (ORIA, Parent-WA44) was installed in each group pen to monitor the environmental temperature and humidity, and the temperature–humidity index (THI) was recorded on an hourly basis. The THI was calculated according to the formula described in Salama et al. [14]:

THI = Td − (0.55 − 0.55 × RH) × (Td − 58)

where Td is the dry bulb temperature (°F) and RH is the relative humidity (%).

### 2.2. Diets

For 54 days (from T0 to T54), the control group (CRT; *n* = 40) of goats were fed the daily total mixed ration (TMR), whereas the treatment group (TRT; *n* = 40) received the diet that was TMR supplemented with 10 g/head/day of microencapsulated organic acids and pure botanicals (OA/PB; containing 16.7% sorbic acid, 25% citric acid, 1.7% thymol, and 1% vanillin in a matrix of hydrogenated fats; Aviplus R, Vetagro S.p.A., Reggio Emilia, Italy). The goats were administered the TMR once daily between 8.00 and 9.00 AM, after the morning milking. The feed was supplied by distributing it along the feeding lane next to each pen. The ration was formulated to cover the goats’ nutritional requirements according to the Institute National de la Recherché Agronomic (INRA, 2018). The chemical analysis of the TMR was performed according to the methods described in the scientific literature [15,16,17]. Briefly, TMR was ground to pass through a 1 mm sieve and stored in an airtight plastic container for subsequent dry matter (DM) (method number 943.01), ash (method number 924.05), CP (method number 954.01), aNDFom (method number 2002.04), and ADF (method number 973.18) determination [18]. The chemical composition of TMR is shown in Table 1.

### 2.3. Blood and Milk Sampling

Blood and milk samples were collected at T0, T27, and T54 during the morning mil-king, which took place between 6:00 and 8:00 AM. For the determination of the hematological parameters, blood samples were collected from the jugular vein into 10 mL vacutainer tubes (Vacutainers, Becton Dickinson) containing ethylenediaminetetraacetic (K3EDTA) acid as an anticoagulant. For the determination of the hematochemical parameters, a second set of blood samples was collected into 10 mL tubes containing a clot activator. The blood samples were stored at 4 °C and immediately transferred to the laboratory to proceed with the analyses as described in Section 2.4. Composite milk samples (pooled from the two hemi-udders) were manually collected for the bacteriological and milk composition assessments. Before milking, the teats were carefully cleaned with cotton wool impregnated with 70% ethanol according to the method described by Ariznabarreta et al. [20]. After discarding the first streams of milk, two composite milk samples were collected into 50 mL Falcon sterile tubes (Falcon Conical Centrifuge Tubes, Tewsbury, MA, USA) from each lactating goat. All the samples were kept at 4 °C from the time of collection. One sample was used for the bacteriological analysis, which was carried out immediately upon arrival at the laboratory as described in Section 2.5. The second sample was used for determining milk composition as described in Section 2.5, which was always performed within 6 h of collection. At the end of each milking procedure, the milk yield (MY, kg) was recorded for each lactating goat, which was assessed using the electronic milk meter present in the milking room. 

### 2.4. Analysis of Hematological and Hematochemical Parameters

Whole blood samples from K3EDTA tubes were immediately processed for the blood counts using an automated blood analyzer (Melet-Schloesing-MS4, Osny, France) and according to the instructions provided by the manufacturer. The parameters analyzed were: erythrocyte count (red blood cell (RBC)); hemoglobin (Hb) concentration; mean corpuscular volume (MCV); the hematocrit (HCT); mean cell hemoglobin (MCH); mean corpuscular hemoglobin concentration (MCHC); platelets (PLT); mean platelet volume (MPV); leukocyte (white blood cell (WBC)), neutrophil (NEU), basophil (BAS), eosinophil (EOS), monocyte (MON), and lymphocyte (LYM) counts. Serum samples were centrifuged at 3500 rpm for 15 min at 20 °C and analyzed using an automated system photometer (I-Lab Aries Chemical Analyzer—Instrumentation Laboratory S.p.A, Werfen Company, Milano, Italy) for total protein (TP), aspartate aminotransferase (AST), alanine aminotransferase (ALT), creatinine (CREA), and blood urea nitrogen (BUN) determinations. The same operator always performed the analysis according to the internal quality control standards.

### 2.5. Analysis of Milk Bacteriological Characteristics and Milk Composition

The bacteriological analysis was performed using 10 microliters (µL) of each milk sample spread on three different agar mediums, specifically: blood agar supplemented with 5% defibrinated sheep blood; Baird Parker agar supplemented with rabbit plasma fibrinogen on MacConkey agar; Tallium Kristalviolette Tossin agar. The plates were incubated at 37 °C for between 24 and 48 h. Suspected colonies were further tested in order to identify the microorganisms. These tests involved the microscopic examination of the colonies after Gram staining, the catalase test, and the API method (API system, BioMerieux, Italy). Isolated bacteria were subsequently tested for antimicrobial susceptibility using the disc diffusion method (Kirby–Bauer test) performed on Mueller–Hinton agar. Moreover, individual milk samples were analyzed using a MilkoScanTM 7 RM (Foss, Denmark) to determine their milk composition (casein, fat, lactose and urea content, milk coagulation index (MCI), somatic cell count, and fat-free dry matter).

### 2.6. Statistical Analysis

Statistical analyses were carried out using the software JMPpro v16 (SAS Institute Inc., Cary, NC, USA). The Shapiro–Wilk test and Levene’s test [21] were performed to test the data for normality and homoscedasticity, respectively. Then, when necessary, somatic cell count data were logarithmically or Box–Cox transformed before further analysis [22]. The THI data were subjected to a bivariate analysis, and the relationships between the variables were investigated using Pearson’s correlations. A linear mixed effects model was constructed considering the following fixed effects: dietary treatment, sampling time, and their interaction. Each goat was then considered to be an experimental unit and used as the random variable for all the analyses, with T0 data used as the covariate. The normality of the data was checked once again for the resulting model residuals. Least squares means were separated using Student’s *t* test adjusted *p*-values when at least one tendency F-test (*p* ≤ 0.10) was detected in the fixed effect interaction term. Finally, the X-test was implemented to study the incidence of bacteria in the milk samples.

## 3. Results

### 3.1. Animals and Environmental Conditions

All the 80 Saneen goats enrolled in the present study remained healthy for the entire length of the experimental trial (from T0 to T54). The results from the bivariate analysis revealed no differences between the environmental data recorded in the two group pens (CTR and TRT), indicating that the enrolled animals were exposed to the same environmental conditions. The mean THI for the period spanning from T0 to T54 was 73.5 ± 3.83 (±SD).

### 3.2. Hematological and Hematochemical Parameters

None of the samples collected contained clots or had hemolyzed. Table 2 reports the hematological and hematochemical mean data for the two dietary group (CRT and TRT) at each sampling time (T0, T27, and T54) and the statistics from the linear mixed effects model considering the effect of the dietary treatment, sampling time, and their interaction. Considering the hematological parameters, the EOS and PLT levels were higher in CRT compared with those in TRT on the basis of the dietary treatment (*p* = 0.01 and *p* < 0.01, respectively). Moreover, the levels of EOS and PLT increased over time in both groups (*p* < 0.01 and *p* = 0.03, respectively). The NEU concentrations also differed between the two groups on the basis of the dietary treatment (*p* = 0.05), with lower values in CRT compared with those in TRT at T27 (45.3% vs. 47.0%, respectively), and the higher values in CRT compared with those in TRT at T54 resulted in a significant interaction between the dietary treatment and sampling time (54.9% vs. 45.7%, respectively; *p* < 0.01). The RBC count varied between the two groups on the basis of the dietary treatment (*p* < 0.01), with there being lower values in CRT compared with those in TRT at T27 (13.7 × 10^6^/µL vs. 13.9 × 10^6^/µL, respectively), and higher values in CRT compared with those in TRT at T54, causing a significant interaction between the dietary treatment and sampling time (13.6 × 10^6^/µL vs. 13.1 × 10^6^/µL, respectively; *p* < 0.01). An effect of the dietary treatment was also seen on the MCH, with the values being higher in CRT than they were in TRT (*p* < 0.01); however, while the mean MCH value increased over the sampling time in CRT, it decreased in TRT (*p* < 0.01), with a significant interaction with the between diet and sampling time at T54 (6.30 pg in CRT vs. 5.50 pg in TRT, *p* < 0.01). The MCHC was influenced by both the dietary treatment (*p* < 0.01) and the sampling time (*p* = 0.04). Specifically, the MCHC values were higher in CRT compared with those in TRT, with a significant interaction between the diet sampling times at T27 (33.0 g/dL vs. 31.2 g/dL, respectively; *p* < 0.01) and T54 (34.5 g/dL vs. 30.7 g/dL, respectively; *p* < 0.01). Differences between the two groups of animals on the basis of diet were also found in relation to HCT (*p* < 0.01), which was lower in CRT than it was in TRT at T27 (24.1% vs. 25.4%, respectively), whereas it was higher in CRT than it was in TRT at T54 (25.2% vs. 24.2%, respectively). The MON and LYM concentrations decreased in both CRT and TRT over the sampling time (*p* < 0.01 and *p* = 0.05, respectively). The Hb values differed between the two groups of animals on the basis of the dietary treatment, with them being higher in CRT than they were in TRT at both T27 (8.10 g/dL vs. 7.90 g/dL, respectively; *p* < 0.01) and T54 (8.25 g/dL vs. 7.35 g/dL, respectively; *p* < 0.01), and with a significant interaction between the dietary treatment and sampling time (*p* < 0.01). Regarding the hematochemical parameters, the CREA level was higher in CRT compared with that in TRT on the basis of the dietary treatment (*p* = 0.01), and the values increased in both groups according to the sampling time (*p* < 0.01). BUN also differed between the CRT and TRT, showing an interaction between the diet and sampling time at T54 (36.7 mg/dL and 34.3 mg/dL, respectively). The TP level was higher in CRT than it was in TRT due to the dietary treatment (*p* = 0.01). Moreover, the TP level increased in both groups according to the sampling time (*p* < 0.01).

### 3.3. Milk Bacteriological Characteristics and Milk Composition

None of the milk samples collected at any of the sampling times (T0, T27, and T54) or from either animal group (CRT and TRT) were found to be contaminated. Table 3 reports the mean milk composition results according to dietary group (CRT and TRT) and sampling time (T0, T27, and T54). MY was influenced by the sampling time (*p* < 0.01): the level of it was lower in both groups with respect to T0 at T27 (2.82 kg in CRT and 2.77 kg in TRT), but then, it increased in both groups at T54 (3.09 kg in CRT and 3.14 kg in TRT). The milk fat content varied significantly according to the dietary treatment (*p* = 0.04), with it being higher in TRT compared with that in CRT at both T27 (2.56% and 2.12%, respectively) and T54 (2.24% and 2.04%, respectively). The MCI was also influenced by the dietary treatment (*p* = 0.03), it was lower in CRT compared with that in TRT at T54 (75.1% and 75.6%, respectively), resulting in a significant interaction between the dietary treatment and sampling time at T54 (*p* = 0.02). The % of lactose differed between the groups according to the sampling time (*p* < 0.01), it was higher in CRT compared with that in TRT at T27 (4.24% and 4.20%, respectively), but it was lower in CRT compared with that in TRT at T54 (4.09% and 4.11%, respectively). The concentration of milk urea also differed between the groups according to the sampling time (*p* < 0.01), with lower values in CRT compared with those in TRT at T27 (26.1 mg/dL and 27.0 mg/dL, respectively), but there were equal values in CRT and TRT at T54 (28.2 mg/dL and 28.2 mg/dL, respectively). The fat-free DM, the FCM, the FPMC, and the ECM were not influenced by the dietary treatment, but they did vary in both CRT and TRT on the basis of the sampling time (*p* < 0.01), showing reduced levels at T27 with respect to those at T0 and higher levels at T54. All the 80 Saneen goats enrolled in the present study remained healthy for the entire length of the experimental trial (from T0 to T54). The results from the bivariate analysis revealed no differences between the environmental data recorded in the two group pens (CTR and TRT), indicating that the enrolled animals were exposed to the same environmental conditions. The mean THI for the period spanning from T0 to T54 was 73.5 ± 3.83.

## 4. Discussion

### 4.1. Animals and Environmental Conditions

To the best of our knowledge, the dietary supplementation of OA (citric and sorbic acids) and PB (thymol and vanillin) microencapsulated in a slow-release lipid matrix (OA/PB) has never previously been investigated in dairy goats. Some studies are available in the scientific literature on other livestock species, for which this specific microencapsulated compound was found to improve both productivity, in terms of growth performances and milk yield, and the physiological and immune statuses [6,11,12]. It is well known that blood and milk parameters in healthy goats can vary in relation to several factors, such as breed, physiological status, and housing conditions [23]. In particular, certain environmental conditions, namely an increase in the temperature–humidity index (THI), can negatively influence the health of ruminant species and their milk production by decreasing the dry matter intake and shifting glucose utilization away from milk synthesis [24]. The scientific literature on the impact of heat stress in dairy goats is sparser than that which is available for dairy cows. In dairy cows, milk production already becomes negatively affected at THI values of 72–75 [25]; goats, on the other hand, are less sensitive to heat stress [26]. The impact of heat stress on dairy goats depends on the breed [27] and on the stage of lactation, with higher milk yield losses in early lactating goats compared with those in mid–late lactating goats [14]. For example, a moderate-to-severe heat stress occurred in dairy Saanen goats following exposure to a THI of 81 or 89 for 4 consecutive days [28] or a THI of 79 for 5 weeks [27]. In the present study, Saanen dairy goats in the mid–late lactation phase received a dietary supplement of OA/PB for 54 days. As the study was carried out during the summer period (July–August), the THI was monitored on an hourly basis throughout the course of the feeding trial (from T0 to T54). The mean ± SD THI recorded was 73.5 ± 3.83, and the results of the bivariate analysis showed no differences between the environmental data recorded in the two group pens (CRT and TRT). These findings indicate that our goats were not exposed to a condition of heat stress at any time during the feeding trial, implying that any differences found in the blood and milk parameters evaluated were related to the dietary treatments and not the environmental factors.

### 4.2. Hematological and Hematochemical Parameters

As previously stated, the effects of OA and PB have been mainly investigated in pigs and poultry, whereas the available information about their supplementation in ruminant feed is still limited. In our study, the challenge diet (TRT) was associated with decreases in the red blood cell (RBC) parameters, namely the RBC counts, HCT, Hb, MCH, MCHC, and PLT. Several stressors have been reported to cause a decrease in the mature RBC counts and the release of immature forms, called reticulocytes, characterized by a lower Hb content, higher volume, and, by consequence, a lower MCHC [2]. The differences between TRT and CRT revealed in the present study are difficult to explain on the basis of the current scientific literature, and although the TRT group had lower RBC parameter values than the CRT group did, the results reflect a physiological rather than a pathological condition since they fell within the normal ranges for healthy goats [29,30]. Moreover, although no differences in the WBC concentration were detected, we did observe changes in specific types of white blood cell, with fewer EOS and NEU in TRT compared with those in CRT due to the dietary supplementation. This finding may suggest that the OA/PB supplementation could also have an immunomodulatory effect on dairy goats, as previously confirmed in non-ruminant species [31]. Concerning the effects of the OA/PB on the hematochemical parameters, the CREA and TP levels were higher in CRT compared with those in TRT as a result of the dietary treatment. TP and blood urea nitrogen (BUN) are indicators of protein metabolism in the organism. Abas et al. [32] found that the supplementation of organic acids in the rations of yearling lambs resulted in higher TP and lower BUN values. No differences in the BUN or milk urea nitrogen concentration (MUN) were found between the two dietary groups in the present study, but the goats fed the TRT diet showed lower values of TP. Unfortunately, it was not possible to calculate the dry matter intake, but on the basis of the available scientific literature, we may speculate that OA/PB supplementation could lead to the more efficient utilization of dietary nitrogen in dairy goats, as previously seen in dairy cows [6]. The increase in the CREA concentration in the blood over the sampling period in both the CRT and TRT groups was most likely derived from a lactation-induced increase in muscle metabolism and is probably related to the normal muscle turnover occurring in mid–late lactation dairy goats [33,34]. Moreover, we found that the CREA concentration was lower in TRT compared with that in CRT, an effect of the dietary treatment. When a marked increase in the CREA concentration is observed, it is generally related to a decline in renal function. However, although the lower CREA levels observed in the goats is an encouraging result, the potential effect of OA/PB improving renal function should be explored in more detail.

### 4.3. Milk Parameters

Although milk yield is expected to decrease over time as per the normal lactation curve for dairy goats [35], no defined trend was observed in the present study, probably because the period between the sampling times was too short to reveal any differences. Nonetheless, in the context of the dairy goat industry, the milk composition and its technological properties are traits of great interest since most of the milk produced is processed into cheese [36,37]. Milk composition can be influenced by several factors such as breed, parity, nutrition, and environmental effects [38]. The prime factor reported to influence milk composition is the presence of subclinical mastitis (followed by the animal being toward the end of the lactation phase) [39]. For this reason, we evaluated the bacteriological characteristics of all the milk samples throughout the feeding trial, and the results confirmed the absence of any bacteriological contamination due to mastitis. Moreover, the milk fat content and milk coagulation index in the TRT group improved over time. Milk fat is an important factor for cheese yield and firmness, as well as in determining the color and flavor of dairy goat products [40]. Lipid supplementation in goat diets is generally associated with a higher milk fat content [41]; however, our findings suggest that supplementation with OA/PB could be useful for increasing the milk fat content in the mid–late lactating phase of dairy goats. This could be due to an increase in fatty acid synthesis in the mammary gland [6,42], but it may also be due to the fact that the supplementation of the total mixed ration with OA positively influences microbial fermentation in the rumen [43], which has been shown to increase volatile fatty acid production and reduce methanogenesis in dairy cows [44]. In fact, milk fat synthesis requires palmitate, which is synthetized from acetate [45,46]. However, the microencapsulated OA/PB mixture should be rumen protected and, accordingly, only exert its effect in the small intestine. However, the mechanism through which the microencapsulated OA/PB exerts its effect on the gut mucosa of ruminants, improving gut health and the intestinal barrier integrity, is uncertain at present [1,6]. Therefore, further studies are required to understand the mode of action of OA/PB in ruminants. Investigations should be directed at elucidating the effect of OA/PB supplementation on the gut microbiota, which is known to influence the gut environment and intestinal permeability [22].

## 5. Conclusions

This is the first study to evaluate the effects of supplementing the dairy goat diet with OA/PB. The hematological and hematochemical parameters revealed no negative consequences of OA/PB supplementation. Moreover, the finding that OA/PB may support renal function is certainly of interest and worthy of further investigation. The results obtained demonstrate that OA/PB exerts positive effects on milk performance by increasing the fat content and the milk coagulation index. Since goat’s milk is mainly used for cheesemaking, these findings encourage further research on OA/PB supplementation in the dairy goat diet.

## Figures and Tables

**Table 1:** Chemical composition of the TMR. Values are expressed as means ± SD.

	TMR ^a^
Dry matter (DM), %	45.0 ± 1.15
Ash, % of DM	8.50 ± 0.25
Crude protein, % of DM	15.8 ± 0.54
Starch, % of DM	21.8 ± 1.71
aNDFom ^b^, % of DM	33.8 ± 1.26
ADF ^c^, % of DM	22.8 ± 0.81
ADL ^d^, % of DM	3.62 ± 0.19
uNDF_240_^e^, % of DM	9.85 ± 0.53

^a^ TMR ingredients: 35.1 % meadow hay silage, 32.4% complementary pelleted feed (containing crude protein 18%, ether extract 5%, crude fiber 8%, crude ash 7.5%), 16.2% second-cut alfalfa hay, 10.8% second-cut meadow hay, and 5.40% wheat bran. The hay was evaluated for its quality and the absence of undesirable weeds [19]. ^b^ Amylase- and sodium sulfite-treated NDF with ash correction. ^c^ Acid detergent fiber. ^d^ Acid detergent lignin. ^e^ Unavailable NDF estimated by 240 h in vitro fermentation.

**Table 2:** Hematological and hematochemical parameters for CRT (control group, *n* = 40) and TRT (treatment group, *n* = 40) blood samples collected at the three sampling times (T0, T27, and T54). Data are expressed as means ± standard error of the mean (SEM).

Item	Diet	Time						*p*-Values			
		T0		T27		T54		Diet	Time	DietxTime	Covariance
		Mean	SEM	Mean	SEM	Mean	SEM				
WBC, 10^3^/µL	CRT	13.8	3.35	12.4	4.10	12.6	5.27	0.71	0.37	0.08	<0.01 *
	TRT	12.2	3.17	11.2	3.29	11.3	3.81				
EOS, %	CRT	6.05	2.60	7.20	2.20	10.9	4.65	0.01 *	<0.01 *	0.12	0.03 *
	TRT	2.60	1.83	6.10	2.88	9.05	2.40				
NEU, %	CRT	49.9	0.94	45.3	1.53	54.9	2.34	0.05 *	<0.01 *	<0.01 *	<0.01 *
						<0.01 *					
	TRT	49.2	0.93	47.0	1.10	45.7	0.88				
BA, %	CRT	0.45	0.50	0.50	0.60	0.40	0.50	0.54	0.29	0.03 *	0.02 *
	TRT	0.40	0.50	0.30	0.50	0.50	0.55				
MON, %	CRT	4.20	0.93	4.00	1.00	3.60	1.38	0.18	<0.01 *	0.13	<0.01 *
	TRT	4.00	0.78	3.40	0.85	3.45	0.80				
LYM, %	CRT	38.8	0.81	42.8	1.43	40.1	1.41	0.41	0.05 *	0.31	<0.01 *
	TRT	41.2	1.01	42.1	1.11	41.2	0.90				
RBC, 10^6^/µL	CRT	13.5	2.04	13.7	2.15	13.6	6.21	<0.01 *	0.69	0.02 *	<0.01 *
						<0.01 *					
	TRT	14.2	1.74	13.9	1.33	13.1	1.79				
MCH, pg	CRT	6.15	0.40	5.90	0.50	6.30	0.75	<0.01 *	<0.01 *	<0.01 *	<0.01 *
						<0.01 *					
	TRT	6.10	0.78	5.70	0.60	5.50	0.88				
MCHC, g/dL	CRT	34.4	0.24	33.0	0.20	34.5	0.36	<0.01 *	0.04 *	<0.01 *	<0.01 *
				<0.01 *		<0.01 *					
	TRT	33.8	0.37	31.2	0.29	30.7	0.37				
MCV, µm^3^	CRT	17.9	1.80	18.0	1.90	18.4	2.30	0.81	0.01 *	0.54	<0.01 *
	TRT	18.1	3.48	18.3	3.15	18.4	3.33				
Hb. g/dL	CRT	8.15	0.95	8.10	1.00	8.25	5.13	<0.01 *	0.63	0.01 *	<0.01 *
						<0.01 *					
	TRT	8.70	0.88	7.90	0.55	7.35	0.80				
HCT, %	CRT	23.5	3.10	24.1	2.50	25.2	13.0	<0.01 *	0.81	0.12	<0.01 *
	TRT	25.6	3.65	25.4	3.50	24.2	4.65				
PLT, 10^3^/µL	CRT	1054	46.0	113	67.0	122	57.3	<0.01 *	0.03 *	0.13	<0.01 *
	TRT	114	82.0	94.5	73.5	99.0	98.0				
CREA, mg/dL	CRT	0.38	0.03	0.26	0.01	0.64	0.02	0.01 *	<0.01 *	0.20	<0.01 *
	TRT	0.47	0.01	0.25	0.01	0.60	0.02				
BUN, mg/dL	CRT	41.3	1.16	33.2	1.21	36.7	1.10	0.85	0.03 *	0.01 *	<0.01 *
	TRT	40.7	1.14	34.6	0.97	34.3	1.18				
TP, g/L	CRT	7.82	0.05	7.87	0.06	7.94	0.08	0.01 *	<0.01 *	<0.01 *	<0.01 *
				<0.01 *							
	TRT	7.65	0.05	7.19	0.17	7.86	0.06				
ALT, units/L	CRT	31.2	0.63	29.3	0.66	29.3	0.64	0.24	0.55	0.47	<0.01 *
	TRT	29.3	0.87	28.8	0.85	28.3	0.84				
AST, units/L	CRT	147	41.3	160	71.0	165	37.3	0.77	0.15	0.68	<0.01 *
	TRT	144.5	46.8	158	41.8	157	34				

* Statistical significance: *p* ≤ 0.05. Abbreviations: WBC, white blood cell; EOS, eosinophils; NEU, neutrophil; BA, basophil; MON, monocyte; LYM, lymphocyte; RBC, red blood cell; MCH, mean corpuscular hemoglobin; MCHC, mean corpuscular hemoglobin content; MCV, mean corpuscular volume; Hb, hemoglobin; HCT, hematocrit; PLT, platelets; CREA, creatinine; BUN, blood urea nitrogen; TP, total protein; AST, aspartate transaminase; ALT, alanine aminotransferase.

**Table 3:** Milk yield and milk composition for CRT (control group, *n* = 40) and TRT (treated group, *n* = 40) at the three sampling times (T0, T27, and T54). Data are normally distributed and are expressed as means ± standard error of the mean (SEM).

Item	Diet	Time						*p*-Values			
		T0		T27		T54		Diet	Time	DietxTime	Covariance
		Mean	SEM	Mean	SEM	Mean	SEM				
MY, kg/milking	CRT	2.98	0.10	2.82	0.10	3.09	0.10	0.75	<0.01 *	0.42	<0.01 *
	TRT	2.94	0.09	2.77	0.12	3.14	0.10				
Total protein, %	CRT	3.15	0.05	3.12	0.05	3.19	0.06	0.37	0.10	0.22	<0.01 *
	TRT	3.12	0.04	3.15	0.06	3.12	0.05				
Casein, %	CRT	2.38	0.05	2.35	0.05	2.41	0.05	0.25	0.13	0.51	<0.01 *
	TRT	2.34	0.03	2.37	0.05	2.35	0.04				
Fat, %	CRT	2.47	0.75	2.12	0.86	2.04	0.71	0.04 *	<0.01 *	0.46	<0.01 *
	TRT	2.71	0.68	2.56	0.34	2.24	0.49				
MCI, %	CRT	75.4	0.37	75.2	0.31	75.1	0.29	0.03 *	0.29	0.03 *	<0.01 *
						0.02 *					
	TRT	75.1	0.23	75.4	0.18	75.6	0.15				
Lactose, %	CRT	4.21	0.03	4.24	0.04	4.09	0.04	0.59	<0.01 *	0.10	<0.01 *
	TRT	4.18	0.03	4.20	0.03	4.11	0.03				
SCC, 10^3^/µL	CRT	356	819	366	702	382	1181	0.94	0.64	0.58	<0.01 *
	TRT	488	769	574	770	697	1026				
MUN, mg/dL	CRT	37.6	1.50	26.1	1.32	28.2	1.14	0.08	<0.01 *	0.32	<0.01 *
	TRT	36.0	1.11	27.0	0.86	28.2	0.96				
fat-free DM, g	CRT	239	71.3	220	68.2	243	59.26	0.23	<0.01 *	0.87	<0.01 *
	TRT	232	60.1	223	61.0	244	60.8				
FCM, kg	CRT	2.61	0.73	2.49	0.70	2.62	0.59	0.14	<0.01 *	0.78	<0.01 *
	TRT	2.63	0.77	2.47	0.68	2.73	0.62				
FPCM, kg	CRT	2.39	0.65	2.22	0.75	2.50	0.60	0.09	<0.01 *	0.65	<0.01 *
	TRT	2.55	0.58	2.29	0.52	2.59	0.62				
ECM, kg	CRT	2.28	0.58	2.11	0.68	2.36	0.62	0.09	<0.01 *	0.68	<0.01 *
	TRT	2.42	0.57	2.18	0.51	2.45	0.58				

* Statistical significance: *p* ≤ 0.05. Abbreviations: MY, milk yield; MCI, milk coagulation index; SCC, somatic cell counts; MUN, milk urea nitrogen; FCM, fat corrected milk; FPCM, fat and protein corrected milk; ECM, energy corrected milk.

## Data Availability

The data that support the findings of this study are available from the corresponding author upon reasonable request.
